# A New Swindle

**Published:** 1870-10

**Authors:** 


### A New Swindle.

### THE UNIVERSITY MEDICINES.

John Smith is ubiquitous—he turns up in the most remote and unheard-of places, and is engaged in all sorts of business. Recently he has taken to humbugging the people, and has chosen a first-rate field in which to work successfully and reap to himself large profits. Would you believe it, John has set himself up in the quack medicine business?—has opened a shop in fact, and intends dealing out cure-all medicines to the waiting thousands, who are only too anxious to avail themselves of John's medical skill. John has located himself in Syracuse this time, and hails from some wonderful institution dubbed the "New York Medical University," which is claimed to be located on the corner of Clinton place, in New York. This establishment, John claims, has cured 26,340 patients in one year! Only think of that, invalids! John has very graciously opened a branch office of this wholesale-curing-establishment at No. 89 Warren street, Syracuse, and offers to cure anybody or anything, by wholesale or retail, who will only send him from five to ten dollars for his UNIVERSAL MEDICINES. John advertises liberally, knows the advantage of printer's ink, and uses it unsparingly. John is a *chemicopathic* physician and surgeon, which shows that he is shrewd in his selection of a name—chemicopathic is good, very good, much better we fear than John's University medicines. John is down on all other kind of doctors, and gives them particular fits in the various "supplements" which he issues to the different country papers throughout the State. This is a good trait in John—it convinces everyone who reads them that John is right and understands his business, for what John can't cure, it is useless for any other member of the Smith family to waste time upon.

It is needless for us to wish John success—he will certainly have it. People *will* be hum bugged, and we suppose John might as well do it, with his "University Medicines," as any one else. It is a first-class way for people to waste money and trifle with disease, and we recommend all in search of such innocent amusement to apply to "Dr. John E. Smith, of the Syracuse wholesale branch of the New York Medical University," and dispenser of the cure-all-heal-over-recuperating-medico-quacko-*chemicopathic* University medicines.

